# Mechanisms by which kidney-tonifying Chinese herbs inhibit osteoclastogenesis: Emphasis on immune cells

**DOI:** 10.3389/fphar.2023.1077796

**Published:** 2023-02-06

**Authors:** Yi Jiao, Xing Wang, Qiong Wang, Qishun Geng, Xiaoxue Cao, Mengxiao Zhang, Lu Zhao, Tingting Deng, Yuan Xu, Cheng Xiao

**Affiliations:** ^1^ Beijing University of Chinese Medicine, China-Japan Friendship Clinical Medical College, Beijing, China; ^2^ Institute of Clinical Medicine, China-Japan Friendship Hospital, Beijing, China; ^3^ Graduate School of Peking Union Medical College, Chinese Academy of Medical Sciences/Peking Union Medical College, Beijing, China; ^4^ China-Japan Friendship Hospital, Capital Medical University, Beijing, China; ^5^ Department of TCM Rheumatology, China-Japan Friendship Hospital, Beijing, China; ^6^ Department of Emergency, China-Japan Friendship Hospital, Beijing, China

**Keywords:** kidney-tonifying Chinese herbs, tonifying kidney-yang herbs, nourishing kidney-yin herbs, osteoclasts, immune cells

## Abstract

The immune system plays a crucial role in regulating osteoclast formation and function and has significance for the occurrence and development of immune-mediated bone diseases. Kidney-tonifying Chinese herbs, based on the theory of traditional Chinese medicine (TCM) to unify the kidney and strengthen the bone, have been widely used in the prevention and treatment of bone diseases. The common botanical drugs are tonifying kidney-yang and nourishing kidney-yin herbs, which are divided into two parts: one is the compound prescription of TCM, and the other is the single preparation of TCM and its active ingredients. These botanical drugs regulate osteoclastogenesis directly and indirectly by immune cells, however, we have limited information on the differences between the two botanical drugs in osteoimmunology. In this review, the mechanism by which kidney-tonifying Chinese herbs inhibiting osteoclastogenesis was investigated, emphasizing the immune response. The differences in the mechanism of action between tonifying kidney-yang herbs and nourishing kidney-yin herbs were analysed, and the therapeutic value for immune-mediated bone diseases was evaluated.

## 1 Introduction

**FIGURE 1:**
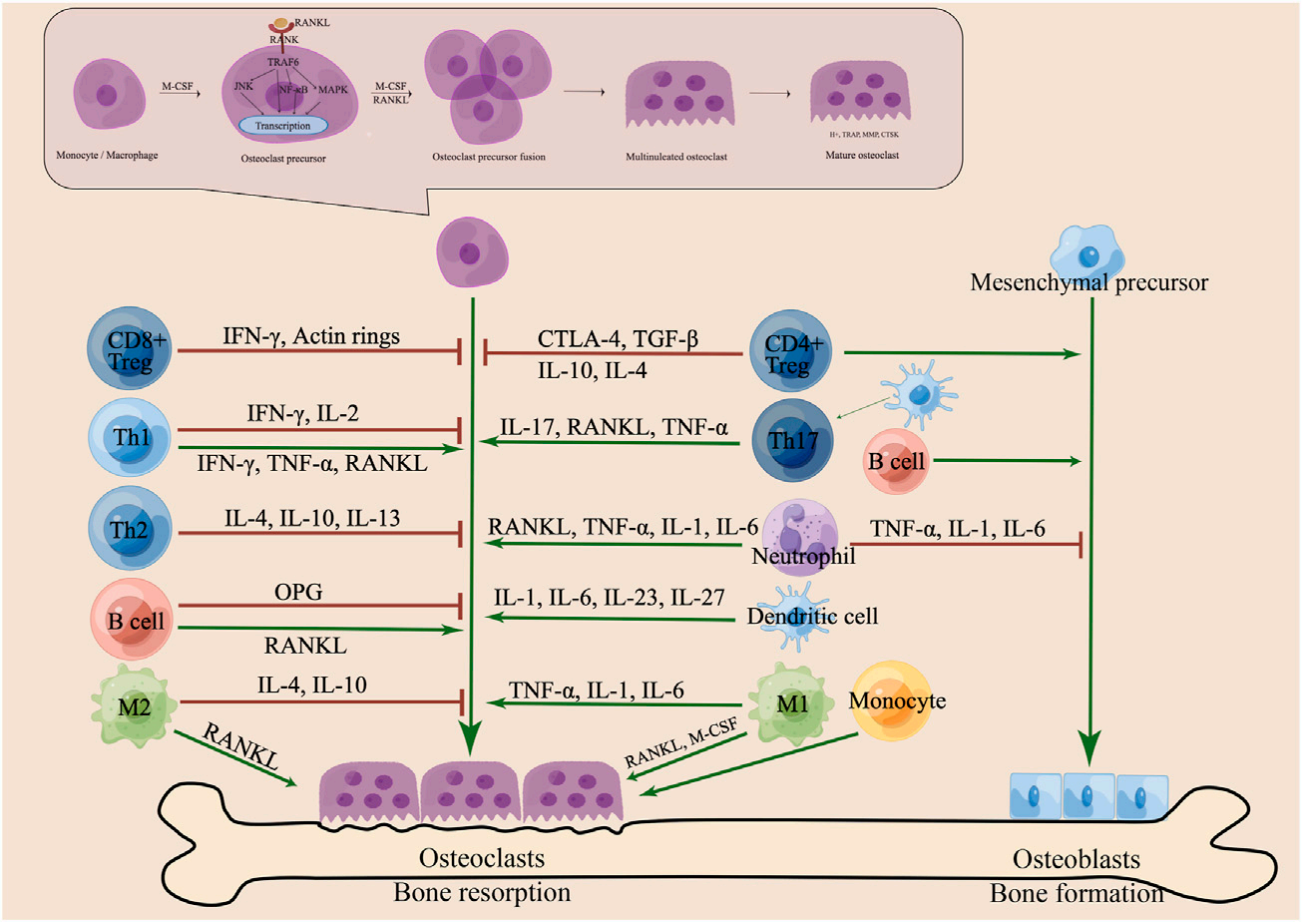
Schematic diagram of the interaction between immune cells and osteoclasts (OCs). Various immune cells and secreted cytokines act on OCs and osteoblasts (OBs) to maintain bone homeostasis on the bone surface. Th-cell subsets (Th1, Th2, Th17, Treg) play an important role in modulating the differentiation and maturation of OCs. Th1 cells secrete IFN-γ, IL-2, TNF-α and RANKL and Th2 cells secrete IL-4, IL-10, and IL-13 to regulate osteoclastogenesis. Th17 cells secrete IL-17, RANKL and TNF-α to enhance OC differentiation and bone resorption. CD4^+^ Tregs promote the bone formation and secrete CTLA-4, TGF-β, IL-10 and IL-4 to promote apoptosis and inhibit bone resorption. CD8^+^ Tregs secrete IFN-γ to inhibit the formation of the actin ring and OC activity. However, B cells secrete OPG and RANKL to regulate bone health. The two subtypes (M1 and M2) of macrophages (Mφs) differentiate into OC and secrete cytokines to regulate osteoclastogenesis. Monocytes (Mo), dendritic cells (DC) and neutrophils and cytokines are associated with OC formation and function. Pre-OCs are derived from monocytes/macrophages and differentiate into mature OCs under stimulation with M-CSF and RANKL. RANKL induces the receptor RANK on the OC surface, which recruits TRAF6 to activate downstream pathways and transcription factors. OC precursors fuse into multinucleated cells and then secrete H+, TRAP, MMP, and CTSK to absorb bone on the bone surface. Pre-OBs are derived from mesenchymal precursors and differentiate into mature OBs, which support bone formation on the bone surface.

Osteoclasts (OCs) originate from the monocyte/macrophage lineage and form large multinucleated cells through cell-cell fusion. Kodama and Kaito, (2020) Osteoclastogenesis is a complex process that requires multiple cytokines, such as receptor activator of nuclear factor (NF)-κB ligand (RANKL), macrophage colony stimulating factor (M-CSF), interleukin (IL), and tumor necrosis factor (TNF), to activate OC differentiation and promote bone resorption. Zhou et al. (2022) M-CSF and RANKL exposure induced the differentiation of OC precursor cells, which were encouraged to proliferate and survive by M-CSF and to differentiate by RANKL; Teitelbaum, (2000) RANKL induces the receptor RANK on the OC surface, which directly recruits tumour necrosis factor receptor-associated factor (TRAF) 6 and activates downstream related signalling pathways, while osteoprotegerin (OPG) competes with RANKL to bind RANK, which induces the activation of various signal transduction pathways, such as the nuclear factor-κB (NF-κB), protein kinase B, PKB (AKT) and mitogen-activated protein kinase (MAPK). Park et al. (2017); Boyle et al. (2003) Nuclear factor of activated T cells (NFAT) c1 and c-Fos act as important transcription factors for OCs, Takayanagi et al. (2002) promoting the expression of genes encoding proteins, such as tartrate-resistant acid phosphatase (TRAP), cathepsin K (CTSK), matrix metallopeptidase (MMP), dendritic cell-specific transmembrane protein (DC-STAMP) and OC stimulatory transmembrane protein (OC-STAMP), to fuse and mature precursor cells. Boyce, (2013) Mature OCs fused by multinucleated cells have folded edges, adhere to the bone matrix, and release protons, chloride and related cathepsins, such as H+, MMP, TRAP, and CTSK, which can effectively degrade collagens and other bone proteins, leading to the dissolution of mineralized matrix; Boyle et al. (2003); Søe et al. (2021) Bone resorption caused by mature OCs on the surface of bone marrow maintains bone homeostasis together with osteoblasts (OBs) that play a role in bone formation. Excessive osteoclastogenesis leads to bone diseases, such as osteoporosis (OP), rheumatoid arthritis (RA), osteosarcoma, and osteopetrosis, (Yao et al., 2021) and disorders of the immune system mediate the process of disease progression; Wu et al. (2021); Andreev et al. (2022); (Figure 1).

Osteoimmunology helps to formulate treatment strategies for various immune-mediated bone diseases involving the bone and the immune systems, and it also highlights the increasingly important role of the immune system in bone health. The topic covers both the indirect relationships between transcription factors, cytokines, and their receptors as well as the direct relationships between the essential cells of the two systems. Okamoto et al. (2017) Through molecular mechanisms, various types of cells in the immune system, including T lymphocytes, B lymphocytes, and macrophages (Mφs), regulate OCs, OBs, chondrocytes, mesenchymal stem cells (MSCs) and other bone cells. Deng et al. (2021) OCs are innate immune cells in bone and have immunological functions in addition to bone absorption; Wu et al. (2008) Its pathological changes are characterized by immunological alterations; Madel et al. (2019) Many studies have proven that T cells and derived cytokines can regulate OCs; Kong et al. (1999); Takayanagi et al. (2000); Sato et al. (2006); Kelchtermans et al. (2009); Levescot et al. (2021) Immune-mediated bone diseases include RA, postmenopausal osteoporosis (PMOP), psoriasis arthritis and ankylosing spondylitis (AS). The infiltration of immune cells and cytokines secreted by immune cells are important mechanisms causing joint deformity and dysfunction. Tang et al. (2020) T-cell-mediated inflammation is an important factor affecting bone mass in PMOP (Wu et al. 2021), and studies have shown that ovariectomy (OVX)-mice lacking T cells have reduced bone loss. Roggia et al. (2001) The regulatory effect of inflammatory factors on MSCs, OBs, and OCs mainly affects the disease progression of PMOP. Ilesanmi-Oyelere et al. (2019); Liao et al. (2020) In the pathogenesis of RA, the inflammatory response disrupts bone homeostasis, leading to bone loss and fractures. Initially, the body develops autoreactive antibodies due to certain factors, followed by autoantibodies, epitopes and inflammatory markers gradually increasing, leading to bone erosion. Finally, immune cells are recruited into the joints, and the inflammatory response is repeatedly cycled and gradually amplified. Andreev et al. (2022) Thus, immune cells and their secreted products in immune-mediated bone diseases activate a variety of pathways, which together comprehensively enhance the differentiation of OCs.

Traditional Chinese medicine (TCM) holds the theory that “kidney governs bone” (Ju et al. 2014), thus, bone diseases are mostly treated from the kidney. Kidney-tonifying Chinese herbs is based on the theory of TCM and has the effect of tonifying the kidney and strengthening the bone, and the common botanical drugs have been tonifying kidney-yang and nourishing kidney-yin herbs according to the medicinal properties and taste since ancient times. Wang et al. (2022b); Zhu et al. (2022) Kidney yang tonifying refers to promoting physiological functions and acting as a facilitator, while kidney yin nourishing refers to providing the subtle material basis for bone conservation, acting as a nourisher. Kidney yang-tonifying herbal medicines include *Epimedium brevicornu Maxim.* (Epimedii Folium), *Morinda officinalis* *F.C. How* (Morindae Officinalis Radix), *Curculigo orchioides Gaertn.* (Curculiginis Rhizoma), *Eucommia ulmoides Oliv.* (Eucommiae Cortex), *Cullen corylifolium (L.). Medik.* (Psoraleae Fructus), *Drynaria roosii Nakaike* (Drynariae Rhizome), *Cnidium monnieri (L.) Cusson* (Cnidii Fructus), while kidney yin-nourishing herbal medicines include *Polygonatum sibiricum Redouté* (Polygonati Rhizome), *Eclipta prostrata (L.) L.* (Ecliptae Herba), *Ligustrum lucidum W.T.Aiton* (Ligustri Lucidi Fructus), *Rehmannia glutinosa (Gaertn.) DC.* (Rehmanniae Radix Praeparata). Zhou and Tang, (2016) Chinese formulae (Han and Kim 2021), botanical drugs (Liu et al. 2017), and natural products (Wang et al. 2018b) used to tonify the kidney have been proven to regulate OCs and OBs and improve bone structure and function to prevent and treat immune-mediated bone diseases with high clinical effectiveness and safety. Kidney-tonifying Chinese herbs have a direct regulatory effect on OCs, but in recent years, based on the close relationship between OCs and the immune system, research into kidney-tonifying herbs controlling OCs *via* the immune system has gained attention. Xia et al. (2021); Zhang et al. (2018); Zhao et al. (2018c) The compound prescription of TCM is the basic form of clinical application in the treatment of immune-mediated bone diseases, and its mechanism of inhibiting osteoclastogenesis through immune cells is relatively clear. But we have limited information on the discussion of the compound prescription of TCM and the differences between the tonifying kidney-yang herbs and nourishing kidney-yin herbs in osteoimmunology. Hence, the review selected the compound prescription, the single preparation and its active ingredients to comprehensively discuss the regulatory mechanism of kidney-tonifying Chinese herbs on OC formation and function from the perspective of immunity, to compare the differences between tonifying kidney-yang and nourishing kidney-yin herbs, and to evaluate the therapeutic value of immune-mediated bone diseases.

## 2 Effects of immune cells on OC formation and function

Since the advent of the field of osteoimmunology, a growing number of studies have focused on the control of OC formation and function through the immune system. OCs are not only phagocytes for bone resorption but are also innate immune cells involved in the control of the immune response. Madel et al. (2019) It was initially believed that the costimulatory signals mediated by immunoreceptor tyrosine-based activation motifs (ITAMs) activated by immune cells were essential for osteoclastogenesis, except for RANKL and M-CSF. Koga et al. (2004) The proliferation, differentiation, fusion, bone adhesion and bone degradation of OCs require the participation of immune cells and secreted cytokines. Del Fattore and Teti, (2012); Madel et al. (2019).

T cells are the core of osteoimmunology. The effect of T cells on osteoclastogenesis depends on the balance of positive and negative factors expressed by T cells. Sato et al. (2006) Only IL-17- expressing Th17 cells have been shown to increase OC formation and bone destruction by promoting the inflammatory response and increasing RANKL expression in OC precursors. In contrast, other T-cell-derived cytokines have a negative effect on osteoclastogenesis. IL-23-stimulated Th17 cells mainly promote osteoclastogenesis by producing IL-17. Sato et al. (2006) IL-17 facilitates local inflammation by recruiting and activating immune cells, resulting in an abundance of inflammatory cytokines, such as TNF-α. Weaver et al. (2006) Inflammatory cytokines enhance RANKL expression, and Th17 cells support osteoclastogenesis through RANKL and CD40L. Gohda et al. (2005) Regulatory T cells (Tregs) secrete M-CSF, transforming growth factor (TGF)-β, IFN-γ, IL-4, IL-10, and IL-5 to inhibit osteoclastogenesis and bone resorption. Kim et al. (2007); Luo et al. (2011); Kelchtermans et al. (2009) A study found that Tregs suppressed the OC differentiation and decreased the areas that were resorbed in cocultures system of Tregs and mouse bone marrow macrophages (BMMS). (Xu et al. 2016b). On the other hand, through CTLA-4 and CD80/86, OC differentiation is inhibited by means of intercellular contact. Axmann et al. (2008) Another study found that CD8^+^ Tregs regulate actin ring formation to inhibit the maturation and activity of OCs *via* IFN-γ. Shashkova et al. (2016) These three mechanisms jointly lead to osteoprotective effects mediated by Tregs. Further studies revealed that gut microbiota and AhR promote the differentiation and function of Tregs to enhance immune tolerance, and that Tregs acted on OCs in both a targeted T cell and antigen-presenting cell (APC) manner. Li et al. (2021)Th1 and Th2 are pioneers in regulating bone health and have bone protection effects in bone immunity. Most cytokines produced by Th1 cells inhibit osteoclastogenesis, including IFN-γ, IL-2, TNF-α, and M-CSF. IFN-γ has both direct anti-osteoclastogenic and indirect pro-osteoclastogenic effects by stimulating T-cell activation and RANKL and TNF-α expression. Gao et al. (2007) TNF-α also has positive and negative effects on the formation of OCs, mainly differentiating M2 macrophages induced by M-CSF into M1 macrophages with the potential to enhance OC formation, but it also inhibits NFATc1 activation and limits RANKL-induced osteoclastogenesis. Zhao et al. (2015) Th2 cells are created by activating CD4^+^ T cells in the presence of IL-4, which causes the production of cytokines. The cytokines IL-4 and IL-13 produced by Th2 cells are associated with the inhibition of osteoclastogenesis and OC differentiation, significantly lowering the RANKL/OPG ratio (Palmqvist et al. 2006), and the cytokine IL-10 is involved in immune inflammatory events in OCs. Gonzalez et al. (2022) New pathways and molecular mechanisms between Th17 cells, Tregs and OCs need to be explored more comprehensively, which is very important for pathological mechanisms and drug research of clinical osteopathy (Figure 1).

Although T cells play a major role in controlling the differentiation of OCs, B cells can also impact problems with OCs, but the regulatory effect of B cells on OCs is still controversial. B cells produce OPG and RANKL to maintain bone homeostasis and support OC differentiation and maturation by regulating the OPG/RANKL/RANK pathway. Grčević et al. (2021); Meednu et al. (2016) On the other hand, B cells act as OC progenitor cells, which may have the potential to produce OCs. Manabe et al. (2001) B cells inhibit OC formation when activated by Th1 cytokines. Choi and Kim, (2003) In turn, OCs regulate the development of B cells by controlling the bone microenvironment and OBs. Mansour et al. (2011) (Figure 1).

The fusion and multinucleation of OCs are attributed to membrane fusion and reprogramming of Mφs. Yao et al. (2021) Mφs, as precursors of OCs, have the potential to differentiate into mature OCs. The two subtypes (M1, M2) differentiate into OC under the conditions of RANKL and M-CSF or RANKL, respectively. Saxena et al. (2021) Oestrogen inhibits M2 polarization mediated by RANKL differentiation into OCs. Dou et al. (2018) This differentiation process is regulated by many factors. Peroxisome proliferator-activated receptor (PPAR)γ, oestrogen-related receptor (ERR)α, PPARγ coactivator (PGC)1β and others have been newly discovered to be involved in OC differentiation in recent years in addition to energy metabolism. Yao et al. (2021) The M1phenotype is an inflammatory that is activated by cytokines, and its related cytokines can promote OC formation and bone resorption, while the M2 phenotype is a repair in which related cytokines, such as IL-4 and IL-10, can inhibit OC function and promote osteogenesis. Xu et al. (2022); Yang and Wan (2019) (Figure 1).

It has been reported that other innate immune cells also have the potential to differentiate into OCs or affect OC formation and function by producing cytokines. Saxena et al. (2021) As the precursor of OCs, monocytes (Mo) are recruited from the blood circulation to the bone surface and differentiate into OCs. Sprangers et al. (2016) Dendritic cells (DCs) differentiate into OCs in an inflammatory state, and DCs act as APCs to activate T cells to promote bone remodelling. Rivollier et al. (2004) Neutrophils, as a type of multinucleated phagocyte, express RANKL to promote bone resorption, while RANK expression depends on TNF-α and IL-4. Poubelle et al. (2007) Infiltration of natural killer (NK) cells in the joint will aggravate the pathological process of RA. Wu et al. (2022a) NK cells mediate the production of RANKL and M-CSF to promote the production of OCs, and induce Mos to differentiate into OCs. Söderström et al. (2010) NK cells inhibit bone destruction through direct contact with OCs or secretion of IL-15. Feng et al. (2015) It shows that NK cells have dual effects on osteoclastogenesis. It can be seen from the above that innate and adaptive immune cells play a key role in the osteoclastogenesis process. Targeting immune cells or related cytokines could inhibit OC formation and function and be used to treat immune-mediated bone diseases. In recent years, the role of N6-methyladenosine (m6A) modification has also been recognized as one of important factors in regulating Mφ and DC activation and function, and T cell homeostasis (Fan et al. 2020), providing a new perspective on osteoimmunology (Figure 1).

## 3 Immunoregulation mechanisms by which tonifying kidney-yang herbs inhibit osteoclastogenesis

### 3.1 The compound prescription of Traditional Chinese medicine

#### 3.1.1 Yi Shen Juan Bi pill

The pill contains botanical drugs including Drynariae Rhizoma, Cistanches Herba and Epimedii Folium, which are used to tonify kidney-yang and strengthen bones. According to the research, Yi Shen Juan Bi pill could affect the activation and differentiation of OCs caused by RA by regulating the phenotypic balance of T cells, upregulating the percentage of Tregs, such as IL-10 and TGF-β1 levels, and downregulating the percentage of Th1 and Th17 cells, such as IFN-γ and IL-17A levels. Wang, (2017); Zhao et al. (2018b) Both *in vivo* and *in vitro* experiments showed that Yi Shen Juan Bi pill directly inhibited the bone resorption function of OCs by inhibiting the JAK2/STAT3 pathway and upregulating the ephrin B2 pathway to downregulate the expression of OC transcription factors, such as c-Fos, c-Jun, NFATc1, RANK, HMGB1 and RAGE. Guo et al. (2018); Cao, (2021); Xia et al. (2021) Further coculture of Tregs and OCs showed that it could also inhibit the JAK2/STAT3 pathway in Tregs to enhance the immunosuppressive effect of Tregs and indirectly inhibit bone resorption function. Xia et al. (2021) Early studies have shown that it can target the inflammatory and immune regulatory responses of Mφs to reduce the production of TNF-α, IL-1 and NO derived from Mφs in the abdominal cavity. Perera et al. (2011).

#### 3.1.2 Bu Shen Tong Luo formula

Drynariae Rhizoma and Epimedii Folium are the most abundant in the formula. The Bu Shen Tong Luo formula could regulate CD3, CD4, and CD8 T cells to improve the joint score of CIA-rats (Su et al. 2010), and it could inhibit the differentiation and maturation of OCs and bone absorption, promote bone reconstruction, alleviate systemic inflammatory response and joint swelling to treat RA and PMOP through the Chemerin, NF-κB, OPG/RANK/RANKL pathways. Han, (2021); Liu et al. (2021a) Its osteoprotective effects may be related to the stimulation of HIF-1α/VEGF angiogenesis. Yuan et al. (2018).

#### 3.1.3 Jia Wei Yang He decoction, Deerhorn Glue pill, Ai Ke Qing granule

The combination of RANKL and M-CSF was used to induce RAW264.7 cells to differentiate into OCs. Cervi Cornu and Epimedii Folium are the botanical drugs to tonify kidney-yang mainly in the three prescriptions. Yang et al. also found that Jia Wei Yang He decoction inhibited OC activity and reduced TRAP activity secreted by OCs through the NF-κB pathway. Yang et al. (2022) suggested that Deerhorn Glue pills could improve bone structure, increase bone strength, inhibit bone absorption and promote OC apoptosis through PI3K/AKT. Yu et al. (2018); Yu et al. (2020) It was found that Ai Ke Qing granules could inhibit OC to treat PMOP, which inhibited the binding of RANK and TRAF6 and affected the activation of downstream NF-κB, p38 and JNK pathways to inhibit functional genes, OC surface receptor genes, OC precursor cell fusion genes, bone resorption functional proteins and transcription factors. Liu (2021) There are many tonifying kidney yang formulae, but they focus on the role of OC differentiation and absorption, and research on immune cells and related cytokines is lacking.

#### 3.1.4 Yi Shen Bu Gu liquid and Bu Shen Qu Han Zhi Wang decoction

**FIGURE 2:**
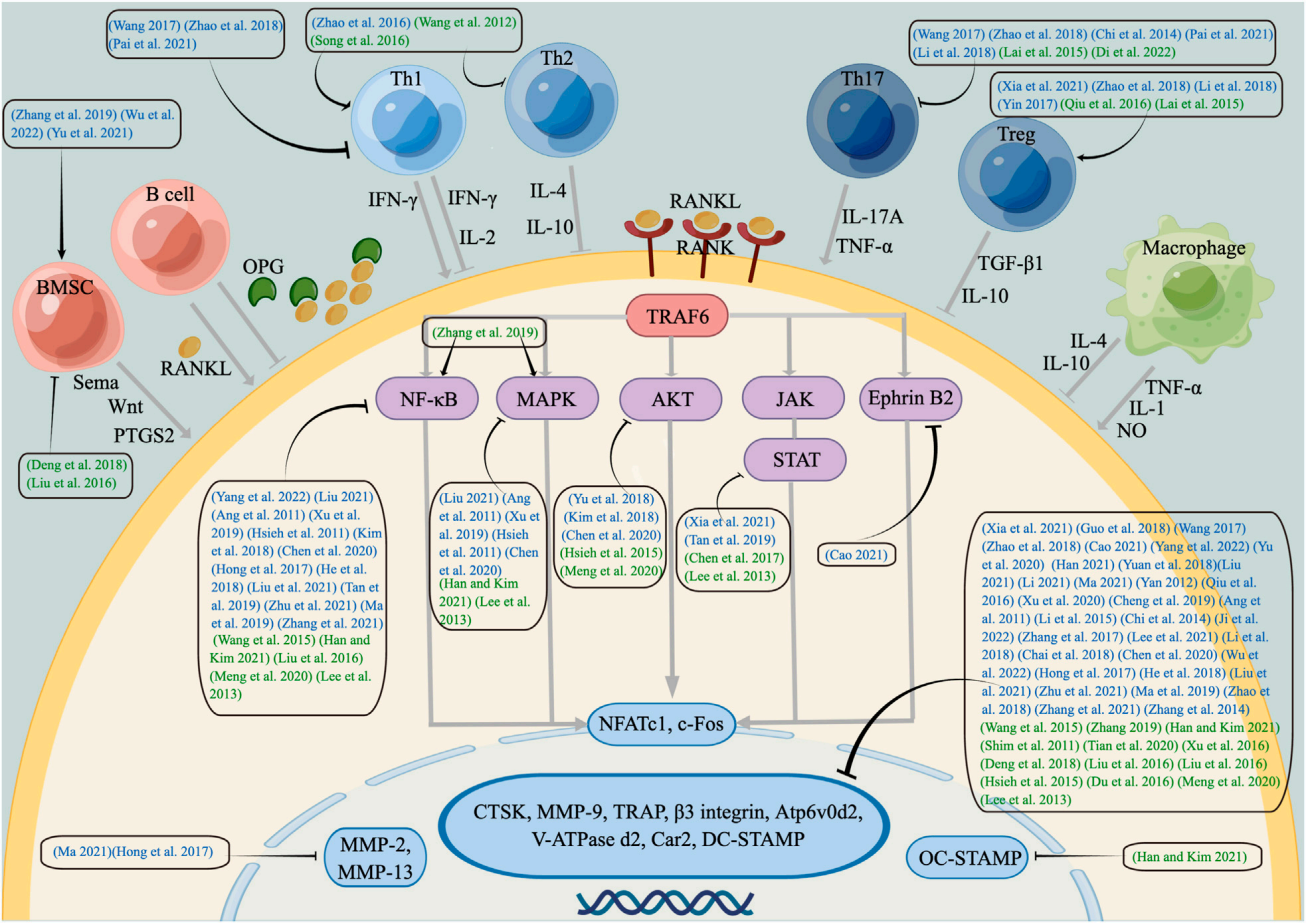
Schematic diagram of immunoregulation mechanisms of kidney-tonifying herbs on inhibiting osteoclastogenesis. Kidney-tonifying herbs inhibit Th1, Th2, Th17, BMSC and related cytokines and activate Th1, Treg, BMSC and related cytokines to suppress the RANKL/RANK, NF-κB, MAPK, AKT, JAK/STAT signalling pathways, transcription factors NFATc1, c-Fos, and OC genes, which play a role in OC formation and function. Blue represents tonifying kidney-yang botanical drugs, while green represents nourishing kidney-yin botanical drugs. Arrows (↓) indicate positive impact, while inverted Ts (⊥) indicate negative impact.

*In vivo* experiments showed that the Chinese herbal prescription for tonifying kidney yang could regulate the inflammatory response, oxidative stress response and downstream pathway through the OPG/RANK/RANKL pathway and play a role in inhibiting OC differentiation. Psoraleae Fructus and Drynariae Rhizoma are the botanical drugs to tonify kidney-yang mainly in the two prescriptions. Yi Shen Bu Gu liquid upregulated IL-33 and downregulated IL-1, IL-7, and TNF-α levels to reduce the inflammatory reaction and increased serum superoxide dismutase (SOD) and catalase (CAT) levels to reduce the oxidation reaction to inhibit OC formation. Li (2021) Bu Shen Qu Han Zhi Wang decoction suppressed OC activity through OPG/RANK/RANKL, reducing the expression of MMP-13, TNF-α, IL-1, IL-2, IL-17, and RANKL to cure RA Li et al. (2017); Ma, (2021); Wu et al.,(2018); Yan, (2012); (Figure 2); (Table 1). These traditional Chinese medicine formulae of tonifying kidney-yang herbs for osteoclastogenesis are preliminarily studied from the cytokines, but the potential immunoregulation mechanisms need to be further explored using immune cells.

**TABLE 1:** Immunoregulation mechanisms by which tonifying kidney-yang of TCM compound prescription inhibit osteoclastogenesis.

Compound prescription	Target factors	Action	Models	Diseases	References
Yi Shen Juan Bi pill	Treg, Th17, Th1 and their cytokines	↑: ephrin B2	OC, OC-Treg coculture, CIA-rats, OVX + CIA-rats, peritoneal macrophages	RA	Xia et al. (2021); Guo et al. (2018); Wang, (2017); Zhao et al. (2018b); Perera et al. (2011); Cao, (2021)
		↓: c-Jun, NO, RANK, JAK2, NFATc1, c-Fos			
Bu Shen Tong Luo formula	CD3 T cell, CD4 T cell, CD8 T-cell and their cytokines	↑: OPG, HIF-1α, VEGF	RAW264.7, CIA-rats, OVX-rats	OP, RA	Han, (2021); Su et al. (2010); Liu et al. (2021a); Yuan et al. (2018)
		↓: RANK, RANKL, Chemerin, p65, TRAP, CTSK			
Yi Shen Bu Gu liquid	Inflammatory cytokines	↑: IL-33, SOD, CAT	OP-mice	OP	Li, (2021)
		↓: IL-1, IL-7, TNF-α, NFATc1, TRAP5			
Bu Shen Qu Han Zhi Wang decoction	Inflammatory cytokines	↑: OPG	CIA-rats	RA	Li et al. (2017); Ma, (2021); Wu et al. (2018); Yan, (2012)
		↓: RANKL, TNF-α, IL-1, IL-2, IL-17, MMP-13			

### 3.2 Single preparation of Traditional Chinese medicine and its active ingredients

#### 3.2.1 Drynariae Rhizoma and its active ingredients

Drynariae Rhizoma, the dried rhizome of Drynaria fortunei J. Sm., is a type of medicine for tonifying kidney yang, and its active substance is mainly naringin, which is widely used in the treatment of OP and fracture. Zhang et al. (2017b) A previous study showed that Drynariae Rhizoma possessed immunoregulatory activity, which stimulated the proliferation of the human osteoprecursor cells, promoted alkaline phosphatase activity and protein secretion, and stimulated cellular and humoral immunity. Jeong et al. (2005) Current studies found that it inhibited the differentiation of Mφs into OC by downregulating the expression of RANKL, NFATc1, Fra-1, Fra-2, c-fos, and CTSK and upregulating the expression of OPG, Wnt10b, and β-catenin. Xu et al. (2020); Cheng et al. (2019); Qiu et al. (2016b); Zhang et al. (2019b) Rhizoma Drynariae Flavonoids positively regulated the immune function of immunocompromised mice by increasing the number of leukocytes in peripheral blood, spleen index, thymus index, the expression of inflammatory factors and the ratio of CD4^+^ T/CD8+ T cell (Zhao et al. 2016), while it regulated the OC function of the OVX rats by regulating the OPG/RANKL/RANK pathway. Song et al. (2016a) Its active substance naringin could inhibit NF-κB activation, IκB-α degradation and ERK phosphorylation and downregulate the expression of related genes induced by RANKL to inhibit the differentiation of Mφs into OCs. Ang et al. (2011); Li et al. (2015) Other studies have found that naringin promotes OCs apoptosis by regulating the mitochondrial apoptosis pathway, downregulating BCL-2, and upregulating Bax, caspase-3 and cytochrome C. Li et al. (2014).

#### 3.2.2 Epimedii Folium and its active ingredient

Icariin is the main active substances in Epimedii Folium, which inhibits the expression of various genes involved in OC formation and bone resorption through the immune response. Icariin reduced Th17 cells by inhibiting STAT3 activation and suppressed IL-17 production, which contributed to the inhibition of cartilage and bone degradation in RA. Chi et al. (2014) *In vitro* experiments showed that icariin inhibited reactive oxygen species and the NF-κB, MAPK and AKT pathways in OCs and promoted negative regulators to inhibit osteoclastogenesis genes and inflammatory factors. Ji et al. (2022); Xu et al. (2019); Hsieh et al. (2011); Li et al. (2022) The results of clinical trials showed that icariin plays an anti-inflammatory role by promoting the apoptosis of CD4^+^ lymphocytes in patients with ankylosing spondylitis. Wang et al. (2017) In addition, icariin decreased inflammatory factor expression to inhibit OC formation in titanium particle-charged calvariae and can be used to treat aseptic loosening after arthroplasty. Shao et al. (2015) Other studies have shown that icariin promoted osteogenic differentiation and inhibited OC differentiation by improving the osteogenic activity of MC3T3-E1 cells, inhibiting the OC activity of RAW264.7 cells, reducing the adipogenic differentiation level of bone marrow-derived mesenchymal stem cells (BMSCs), and blocking the osteoclastogenesis induced by MCF7 and MDA-MB-231 breast cancer cells. Kim et al. (2018); Zhang et al. (2017a).

#### 3.2.3 Psoraleae fructus and its active ingredients

The active substances of Psoraleae Fructus are neobavaisoflavone and psoralen. Psoraleae Fructus, as a Chinese botanical drug for treating RA, reduced the percentages of CD4^+^ IL-17A+, CD4^+^ TNF-α+, and CD4^+^ IFN-γ+ cells in the spleen and the expression of inflammatory factors by increasing myeloid-derived suppressor cells (MDSCs) (Pai et al. 2021), four of which can inhibit RANKL-induced osteoclastogenesis. Lee et al. (2021) Psoralen promoted the balanced development of CD4^+^ CD25^+^ Treg/Th17 to CD4^+^ CD25^+^ T cells by promoting Foxp3, TGF-β and IL-10 levels and inhibiting RORγt, IL-17 and TNF-α levels in CD4^+^ T cells to downregulate OC differentiation-related genes through AP-1 pathways (Li et al. 2018; Chai et al. 2018), and reduced the expression of IL-8 and PTHrP to inhibit the interaction between OCs, OBs and cancer cells and to reduce the burden of bone metastasis caused by breast cancer. Wu et al. (2013) Neobavaisoflavone, another potential bioactive compound, was proven to inhibit osteoclastogenesis and OC function *in vitro* and *in vivo*. Neobavaisoflavone was found to block NFATc1 nuclear translocation and inhibit the activation of the NF-κB, MAPK and AKT pathways and the expression of MMP9, CTSK, CTR and TRAP by disrupting the recruitment of TRAF6 and c-Src molecules by the RANK receptor at the cell membrane. Chenet al. (2020a).

#### 3.2.4 Curculiginis Rhizoma and its active ingredient

Curculigoside is an important active substance of Curculiginis Rhizoma, which is used to treat OP, arthritis and osteolysis. *In vivo* studies have found that curculigoside reduced the spleen and thymus indices and the expression of inflammatory factors in the serum of CIA rats, such as TNF-α, IL-1β, IL-6, IL-10, IL-12 and IL-17A, while inhibiting the expression of inflammatory factors in Ti-induced osteolysis model mice and restoring the RANKL/OPG ratio. The results showed that curculigoside inhibited osteoclastogenesis and alleviated oxidative stress by activating Nrf2 and inhibiting the NF-κB pathway, alleviated the inflammatory response in MH7A cells through the JAK/STAT/NF-κB pathway, alleviated the expression of inflammatory factors and OC genes in BMSCs, and weakened mitochondrial damage in MC3T3-E1 cells. Liu et al. (2021a); Zhu et al. (2021); Tan et al. (2019) In addition, studies have shown that curculigoside can reverse the immunosuppression caused by cyclophosphamide and proinflammatory cytokines in serum and has anticancer potential against cancer cell lines, such as HepG2, HeLa and MCF-7 (Murali and Kuttan 2015; Hejazi et al. 2018), which may have certain therapeutic value for bone metastasis after the development of a tumour.

#### 3.2.5 Morindae officinalis radix and its active ingredients

Morinda officinalis polysaccharide, bajijiasu and rubiadin-1-mrthyl ether are the active components of Morindae Officinalis Radix, which is commonly used to treat RA and OP and has obvious therapeutic effects on senile, postmenopausal, and glucocorticoid osteoporosis. Morinda officinalis could improve atrophy of the spleen and thymus by increasing the number of CD4^+^ cells and reducing the number of CD8^+^ cells in the peripheral blood of immunosuppressive mice induced by cyclophosphamide. Wei et al. (2019) Morinda officinalis polysaccharide could enhance the immune and anti-inflammatory function of psoriasis patients, enhance the growth capacity of Tregs, and weaken immune and proinflammatory functions. Yin, (2017) It protected bone loss and biomechanical dysfunction in OVX-rats and had antiosteoporosis activity through OPG/RANK/RANKL (Zhang et al. 2020; Peng and Zhong 2020), while inhibiting OC differentiation through the exosomes of mesenchymal stem cells, which was closely related to the expression of PTGS2 and miR-101-3p. Wu et al. (2022c) Bajijiasu and Rubiadin-1-mrthyl ether inhibited the expression of OC-specific genes induced by RANKL through the NF-κB pathway to inhibit OC generation and function and treat osteolytic bone diseases. Hong et al. (2017); He et al. (2018).

#### 3.2.6 Other single botanical drugs and their active ingredients

There are other tonifying kidney-yang botanical drugs with the functions of tonifying the kidney, strengthening the yang and strengthening the muscles and bones, which are often used to treat immune-mediated bone diseases, but the mechanism of action on immunity needs to be further studied. Eucommiae Cortex can be used to treat OP and RA, and its active components include polysaccharides, iridoid glycosides, and flavonoids. It was found that ethanol extracts from Eucommiae Cortex alleviated the inflammatory response through the OPG/RANK/RANKL and NF-κB pathways to delay the destruction of intraarticular cartilage and bone. Zhang et al. (2021); Wang et al. (2022a); Zhang et al. (2014) The ethanol extracts and polysaccharides from Eucommiae Cortex increased the coefficient of the thymus and spleen and improved the clearance ability and phagocytosis ability of peritoneal Mφs in mice. Qiu et al. (2008); Ye et al. (2015) Osthole may restore BMSC-mediated T-cell migration and apoptosis by stimulating the expression of Fas/FasL, enhancing the immune activity of BMSCs in healthy or inflammatory microenvironments to treat OP (Yu et al. 2021), and downregulating c-Scr, β3-integrin and OC genes to inhibit OC formation and bone resorption. Ma et al. (2019); Zhao et al. (2018a); (Figure 2); (Table 2).

**TABLE 2:** Immunoregulation mechanisms by which tonifying kidney-yang of single preparations and their active ingredients inhibit osteoclastogenesis.

Single preparation	Active ingredients	Target factors	Action	Models	Diseases	References
Drynariae Rhizoma	Rhizoma Drynariae Flavonoids	Thymus indexes, spleen indexes, T-bet, GATA-3, Inflammatory cytokines	↑: OPG	cyclophosphamide-induced immunosuppression-mice, OVX-rats	OP	Zhao et al. (2016); Song et al. (2016a)
			↓: RANK, RANKL			
Epimedii Folium	Icariin	Apoptosis of CD4^+^ T cell, Th17	↑: OPG	Human periodontal ligament cells, RAW264.7, BMMs, osteolysis model-rats, CIA mice, ankylosing spondylitis patients	Periodontitis, OP, RA, Aseptic loosening, ankylosing spondylitis	Chi et al. (2014); Ji et al. (2022); Xu et al. (2019); Hsieh et al. (2011); Kim et al. (2018); Zhang et al. (2017a); Shao et al. (2015) Wang et al. (2017)
			↓: RANKL, RANK, TRAF6, p-p38, p-JNK, p-ERK, AKT, NFATc1, c-Fos, CTSK, TRAP, MMP-9, ATP6v0d2, β3 integrin, CTR, CK, F-actin			
Psoraleae Fructus	Psoraleae Fructus	Percentages of CD4^+^ IL-17A+, CD4^+^ TNF-α+, CD4^+^ IFN-γ+ cells	↓: RANKL, CTSK	CIA-mice, BMMs	RA, OP	Pai et al. (2021); Lee et al. (2021)
Psoraleae Fructus	Psoralen	Foxp3, RORγt	↑: OPG	BMMs, RAW264.7 and CD4^+^ T-cell coculture, MDA-231BO cells	OP, bone metastases of breast cancer	Li et al. (2018); Wu et al. (2013); Chai et al. (2018)
			↓: RANKL, M-CSF, c-Jun, TRAP, CTSK, MMP-9, PTHrP			
Morindae Officinalis Radix	Morinda officinalis polysaccharide	CD4^+^ T cell, CD8^+^ T cell, Treg	↑: TLR3, miR-101-3p, OPG	BMMs, BMSCs-exosomes, adipose mesenchymal stem cells of psoriasis patients, OVX-rats	OP, psoriasis patients	Yin (2017); Wu et al. (2022c); Zhang et al. (2020); Peng and Zhong (2020)
			↓: RANK, RANKL, TLR4, PTGS2			
Curculiginis Rhizoma	Curculigoside	STAT3	↑: OPG	RAW264.7, fibroblast-like synoviocyte MH7A cells, BMSCs, CIA-rats, Ti induced osteolysis-mice	OP, RA, osteolysis	Liu et al. (2021b); Tan et al. (2019); Zhu et al. (2021)
			↓: RANKL, JAK1, JAK3, p-p65, F-actin, NFATc1, c-Fos, TRAP, CTSK, MMP-9			
Fructus Cnidii	Osthole	CD3^+^ T cell	↓: RANKL, p65, NFATc1, c-Fos, CTSK, MMP9, TRAP, Car1, c-Scr, β3 integrin	BMMs, RAW264.7, BMSCs, T cells	OP	Ma et al. (2019); Zhao et al. (2018a); Yu et al. (2021)
Eucommiae Cortex	Ethanol extracts	Thymus coefficient, spleen coefficient, IL-17, Inflammatory cytokines	↑: OPG	CIA-rats, cyclophosphamide-induced immunosuppression-mice, OVX-rats	RA, OP	Zhang et al. (2021); Qiu et al. (2008); Zhang et al. (2014); Wang et al. (2022a)
			↓: RANKL, p-p65, NFATc1, c-Fos, TRAP, CTSK			

## 4 Immunoregulation mechanisms by which nourishing kidney-yin herbs inhibit osteoclastogenesis

### 4.1 The compound prescription of Traditional Chinese medicine

#### 4.1.1 Bu Shen Ning Xin decoction

Rehmanniae Radix Praeparata is the main nourish kidney-yin component of the decoction. The Bu Shen Ning Xin decoction can treat PMOP by regulating osteoclastogenesis. A previous study suggested that Bu Shen Ning Xin decoction abrogated RANKL-mediated NFATc1 and NF-κB pathways through selective oestrogen receptor (ER)α. Wang et al. (2015) Later, CD4^+^ T cells were cocultured with BMMs to investigate the immune modulatory effects of Bu Shen Ning Xin decoction on CD4^+^ T cells and osteoclastogenesis, and the findings suggest that Bu Shen Ning Xin decoction has an immune-regulating effect on the bone phenotype of OVX mice by changing the immune environment, increasing CTLA-4+ Tregs and the apoptosis rate of CD4^+^ T cells and regulating the RANKL/OPG imbalance in CD4^+^ T cells. Qiu et al. (2016a); Zhang et al. (2018).

#### 4.1.2 Zuo Gui Wan

Rehmanniae Radix Praeparata is the main nourish kidney-yin component of the Zuo Gui Wan, which prevented bone loss by downregulating IL-6 and RORγt, upregulated Foxp3 in OVX mice and skewed the Th17/Treg paradigm to Treg (Lai et al. 2015), which may be related to the OPG/RANKL pathway mediated by β2-adrenergic receptor (β2AR). Liu et al. (2018) Further experimental results show that Zuo Gui Wan inhibited bone resorption by inhibiting OC proliferation and differentiation and promoting OC apoptosis. Zhang, (2019).

#### 4.1.3 Yukmijihwang-Tang

Rehmanniae Radix Praeparata is the main nourish kidney-yin component of the Yukmijihwang-Tang, which inhibited RANKL-induced OC differentiation, resorption and fusion and OC differentiation in the coculture system of bone marrow cells (BMCs) and OBs and BMMs and downregulated OC-related genes, which may be related to the inhibition of the p38, JNK, ERK, and NF-κB signalling pathways. Han and Kim, (2021); Shim et al. (2011) Moreover, the study found that it upregulated the expression of the immune-related gene IRF1 in the JAK/STAT pathway in PMOP patients with kidney yin deficiency. Chen et al. (2017) However, its mechanism has not been explored with regard to immune cells.

#### 4.1.4 Erzhi formula

Ligustri Lucidi Fructus and Ecliptae Herba make up Erzhi formula, which is used to treat kidney yin deficiency. Erzhi formula improved PMOP by controlling the OPG/RANK/RANKL signalling axis, and suppressing the expression of OC differentiation genes, such as CTSK, TRAP and NFATc1, which may be related to inhibiting cell autophagy, controlling oestradiol, and nourishing the uterus. Qin et al. (2021); Tian et al. (2020) Meanwhile, Erzhi formula had a protective effect on immunocompromised mice and could improve the activity level of T lymphocyte factor. Yao et al. (2014) At present, there is only network pharmacological analysis of the Erzhi formula in RA, and basic research to prove the relevant mechanism is lacking. (Figure 2); (Table 3).

**TABLE 3:** Immunoregulation mechanisms by which nourishing kidney-yin of TCM compound prescription inhibit osteoclastogenesis.

Compound prescription	Target factors	Action	Models	Diseases	References
Bu Shen Ning Xin decoction	CTLA-4+ Tregs	↑: OPG	OVX-mice, CD4^+^ CD25^+^ T cell, BMM-CD4^+^ T cell coculture system	OP	Zhang et al. (2018); Wang et al. (2015); Qiu et al. (2016a)
		↓: RANKL, NF-κB, NFATc1			
Zuo Gui Wan	Foxp3, RORγt	↑: OPG	OVX-mice, RAW264.7	OP	Lai et al. (2015); Liu et al. (2018); Zhang (2019)
		↓: TRAP, RANKL, β2AR, NFATc1			
Yukmijihwang-Tang	Inflammatory cytokines	↑: OPG	BMCs and OBs coculture system, BMMs, OVX-rats, human peripheral blood leukocytes	OP	Han and Kim (2021); (Shim et al. (2011); Chen et al. (2017)
		↓: RANKL, p-p38, p-JNK, p-ERK, p-p65, NFATc1, c-Fos, TRAP, Atp6v0d2, OSCAR, OC-STAMP, DC-STAMP, MMP-9, CTSK			
Erzhi formula	Inflammatory cytokines	↑: OPG	OVX-mice, OP-zebrafish, RAW264.7, cyclophosphamide-induced immunosuppression-mice	OP	Qin et al. (2021); Tian et al. (2020); Yao et al. (2014)
		↓: RANK, RANKL, NFATc1, TRAP, CTSK			

### 4.2 Single preparation of Traditional Chinese medicine and its active ingredients

#### 4.2.1 Ecliptae Herba and its active ingredient

Ligustri Lucidi Fructus and Ecliptae Herba are two single botanical drugs of Erzhi formula. Wedelolactone is the active ingredient of Ecliptae Herba. Wedelolactone inhibited the production of Sema3A, Sema4D and Sema7A and the number of Sema4d-plexinB1 and plexinA1-DAP12 complexes in BMSCs, and inhibited RANKL-induced F-actin ring formation and bone resorption pits to inhibit osteoclastogenesis and bone resorption. Deng et al. (2018); Liu et al. (2016a); Liu et al. (2016b) Wedelolactone inhibited breast cancer-mediated osteoclastogenesis and OC activity in CD14^+^ monocytes through the AKT-mTOR pathway to treat bone metastasis after breast cancer. Hsieh et al. (2015).

#### 4.2.2 Ligustri Lucidi Fructus and its active ingredient

Studies have shown that Ligustri Lucidi Fructus could upregulate the gene expression of the T-cell receptor signalling pathway. Zheng et al. (2018) The extract of Fructose Ligustri Lucidi upregulated the levels of IL-2 and IFN-γ produced by Th1 lymphocytes, and downregulated the level of IL-10 produced by Th2 lymphocytes (Wang et al. 2012), while it inhibited OC differentiation and bone resorption by inhibiting RANKL-induced OC formation and TRAP activity and reduced OC differentiation genes in RAW264.7 cells. Xu et al. (2016a).

#### 4.2.3 Polygonati Rhizome and its active ingredient

Polygonatum sibiricum polysaccharide are some of the main components of Polygonati Rhizome. Polygonatum polysaccharide promoted the development of immune organs, lymphocyte proliferation, natural killer cell (NK) activity and phagocytosis of Mφs, increased the ratio of CD4+/CD8+, and mediated the release of cytokines at the cellular and transcriptional levels to reverse immunosuppression (Chen et al. 2020b; Zhang et al. 2019a), and it inhibited OC differentiation in BMMs through the Hippo signalling pathway based on miR-1224 and maintained bone mass in LPS-induced skull osteolysis-mice. Du et al. (2016); Li et al. (2019).

#### 4.2.4 Rehmanniae Radix Praeparata and its active ingredient

Two polysaccharides in Rehmanniae Radix Praeparata promoted phagocytosis of RAW264.7 cells and increased lysozyme activity in a dose-dependent manner. Zhou et al. (2021) At the same time, they have immune activity by promoting the activation of human dendritic cells and T cells and inducing the expression of costimulatory molecules and the production of proinflammatory cytokines. Wang et al. (2018a) Catalpol extracted from Rehmanniae Radix Praeparata upregulated the activity of phosphatase and tensin homologue (PTEN) by reducing ubiquitination and degradation, subsequently suppressing the NF-κB and AKT pathways induced by RANKL but had no effect on the MAPK pathway, leading to an inhibition of OC activity to avoid bone loss. Meng et al. (2020) Its immune mechanism may be related to the fact that catapol could inhibit the traditional differentiation and inflammatory factors of Th17 and inhibit the transdifferentiation of Treg-to-Th17 cells associated with STAT3 by upregulating let-7g-5p. Di et al. (2022) Acteoside is the active substance of Rehmanniae Radix Praeparata, which downregulated NFATc1, c-Fos, TNF-α, IL-6 and ROS in Mφs through the RANKL downstream pathway to inhibit osteoclastogenesis. Lee et al. (2013) Studies have shown that acteoside suppresses the differentiation of macrophage-like cells induced by IL-32 and the inflammatory response through the JAK/STAT pathway. Nam et al. (2015); Qiao et al. (2019) The immune regulation mechanism may selectively enhance the production of IFN-γ by T cells and promote the production of IL-10 derived from B cells by regulating the TLR4/PI3K pathway. Wu et al. (2022b); Song et al. (2016b); (Figure 2); (Table 4).

**TABLE 4:** Immunoregulation mechanisms by which nourishing kidney-yin of single preparations and their active ingredients inhibit osteoclastogenesis.

Single preparation	Active ingredients	Target factors	Action	Models	Diseases	References
Ligustri Lucidi Fructus	Ethanol extract	Inflammatory cytokines	↓: RANKL, c-Fos, NFATc1, TRAP, CTSK, MMP-9, TRAF-6	RAW264.7, blood peripheral lymphocytes	OP	Xu et al. (2016a); Wang et al. (2012)
Polygonati Rhizome	Polysaccharide	Thymus index, spleen index, macrophage phagocytosis, NK cell activity, CD4+/CD8+ ratio	↑: NF-κB, p-p38	RAW264.7, BMMs, LPS induced skull osteolysis-mice	OP	Chen et al. (2020b); Zhang et al. (2019a); Du et al. (2016); Li et al. (2019)
			↓: TRAP, MMP-9, CTSK, NFATc1			
Rehmanniae Radix Praeparata	Catalpol	Tregs, Th17 and their cytokines	↑: PTEN, let-7g-5p	BMMs, RAW264.7, OVX-mice, LPS-mice, CIA-mice	OP, RA	Meng et al. (2020); Di et al. (2022)
			↓: p-p65, p-AKT, p-GSK3β, NFATc1, TRAP, CTSK, DC-STAMP, CTR, V-ATPase d2			
Rehmanniae Radix Praeparata	Acteoside	STAT3	↓: p-p38, ERK, p-JNK, p-p65, p-JAK, NFATc1, c-Fos	BMMs, RAW264.7, THP-1 cells, OA-rats	OP, OA	Lee et al. (2013); Nam et al. 2015); Qiao et al. (2019); Wu et al. (2022b); Song et al. (2016b)

## 5 Conclusion and prospects

Remarkable progress has been made in understanding the regulatory mechanisms of kidney-tonifying herbs on OCs based on the immune system. Through the repeated *in vivo* and *in vitro* experiments collected in this study, the involved immune cells and signalling pathways of the anti-OC effect in tonifying kidney-yang herbs are understood to be more extensive. By regulating Th1, Th2, Th17, Treg, Mφ, and BMSC cells and secreted cytokines and based on the RANK/RANKL signalling pathway, tonifying kidney-yang and nourishing kidney-yin herbs regulate the surface precursor receptors, downstream signalling pathways, transcription factors and OC genes to inhibit OC generation and differentiation. Based on the above, the difference between kidney-yang and kidney-yin herbs is that kidney-yang herbs regulate MMP-2 and MMP-13 genes and have more advantages in treating osteolytic disease and posttumor bone metastases; the kidney-tonifying herbs regulate the OC-STAMP gene and have more advantages in the treatment of estrogen-related diseases, but the differences in the regulation of immune cells are not obvious. Screening for kidney-tonifying herbs with OC biological effect-based immune responses should be further carried out due to the complexity of the active components of botanical drugs. The compound prescription of TCM should receive more consideration as the basic form of clinical application. However, the selected original articles have certain limitations, such as lacking research on the drug dose range, lacking co-culture system for cell models, which lack innovation and guidance for further research. Importantly, the difference between tonifying kidney-yang herbs and nourishing kidney-yin herbs needs to be demonstrated from the perspective of epigenetics and using cell co-culture technology, which may be evidence for the classification basis of botanical drugs and provide additional evidence for selecting more targeted botanical drugs in the clinic.
